# Correlation between estimated plasma remnant-like particle cholesterol and vegetable fat intake in Uku town, Japan

**DOI:** 10.1186/s12199-021-01005-4

**Published:** 2021-08-24

**Authors:** Hisashi Adachi, Tatsuyuki Kakuma, Mika Enomoto, Ako Fukami, Sachiko Nakamura, Yume Nohara, Nagisa Morikawa, Akiko Sakaue, Maki Yamamoto, Yoshihiro Fukumoto

**Affiliations:** 1 Department of Internal Medicine, Division of Cardio-Vascular Medicine, Kurume University School of Medicine, Kurume, 830-0011 Japan; 2 Department of Community Medicine, Kurume University School of Medicine, 67 Asahi-machi, Kurume, 830-0011 Japan; 3 Biostatistics Center, School of Medicine, Kurume University, Kurume, Japan

**Keywords:** Remnant-like particle cholesterol, Lipoproteins, Lipid profile, Vegetable fat, Epidemiology

## Abstract

**Background:**

Remnant-like particle cholesterol (RLP-C) is highly atherogenic, which is associated with atherosclerosis. However, RLP-C has not been routinely measured in the clinical practice. We estimated RLP-C levels using conventional lipid profiles and examined the association between estimated RLP-C and related factors including nutrient intake.

**Methods:**

This study was performed in Uku town, Nagasaki prefecture, Japan in 2019. A total of 225 subjects were enrolled and directly measured RLP-C levels. Estimated RLP-C levels were defined as the following formula [total cholesterol − (LDL-cholesterol) − (HDL-cholesterol)]. Multivariate analyses were used to assess the relationship between estimated RLP-C and atherogenic factors. We calculated cut-off values on dichotomized RLP-C (< 7.5 mg/dL vs. ≥ 7.5 mg/dL) by receiver operating characteristic (ROC) curve.

**Results:**

The mean values of directly measured RLP-C levels and estimated RLP-C were 4.0 mg/dL and 16.4 mg/dL, respectively. In the multiple stepwise linear regression analysis, directly measured and estimated RLP-C levels were independently and commonly associated with apolipoprotein E, triglycerides, and vegetable fat intake (inversely). Using ROC curves, we found the cut-off value of estimated RLP-C was 22.0 mg/dL.

**Conclusion:**

We demonstrated that the estimated RLP-C levels using conventional lipid profiles may substitute for directly measured RLP-C and these levels were independently and inversely associated with vegetable fat intake in the community-dwelling Japanese population.

## Background

Accumulating evidence in epidemiological studies has indicated that remnant-like particle cholesterol (RLP-C) derived from very low-density lipoprotein (VLDL) and/or chylomicron has been progressively recognized as a stronger atherogenic factor than cholesterol [1, 2], and then, RLP-C has been highlighted as a novel risk factor for coronary artery disease [3]. Further, there are several reports dealing with the important association between RLP-C and apolipoproteins [4, 5]. Nevertheless, the levels of RLP-C have not been routinely measured, especially in the clinical practice because of the higher cost than low-density lipoprotein cholesterol (LDL-C) or triglycerides, as well as the limited health care services. Thus, a simple formula is currently used to measure RLP-C cut-off values using total cholesterol, LDL-C, and high-density lipoprotein cholesterol (HDL-C) [6]. Namely, we examined whether estimated RLP-C levels using conventional lipid profiles may substitute for directly measured RLP-C.

On the other hand, it has been also reported regarding RLP-C and nutrient intake [7–9]. However, there has been few studies, which examined the association between estimated RLP-C and nutrients intake. Earlier studies demonstrated that vegetable fat such as soy protein isolate intake reduced remnant lipoproteins [10, 11].

Although our cohort is a small fishing community in Uku town, Japan, the eating pattern in the subjects of the present study was similar to that reported in the results of the National Nutrition Survey [12]. In this circumference, this study was designed to investigate a relationship between directly measured or estimated RLP-C and long-term vegetable fat intake in the community-dwelling Japanese population.

## Methods

### Subjects

A total of 225 subjects (94 males and 131 females: aged 51 to 85 years) received a population-based health examination in Uku town, a fishing community in southwestern Japan in 2019. This town is an isolated island in Sasebo city, located in Nagasaki prefecture, and the total population is about 2100. A detailed content of the recent survey in the same district was previously described [13, 14].

### Data collection

Height and weight were measured, and body mass index (BMI) was calculated as weight (kg) divided by the square of height (m^2^) as an index of the presence or absence of obesity. Waist circumference was measured at the level of the umbilicus in a standing position. Blood pressure (BP) was measured in the right arm twice with a mercury sphygmomanometer after the subject had rested in the sitting (first) and supine (second) position for more than 5 min. Vigorous physical activity and smoking were avoided for at least 30 min before BP measurements. The second BP with the fifth phase diastolic pressure was used for analysis.

Blood was drawn from the antecubital vein for determinations of lipids profiles (total cholesterol, LDL-C, HDL-C, triglycerides, RLP-C, and lipoprotein(a) [Lp(a)]), apolipoproteins (apo A-I, apo B, apo C-III, and apo E), blood urea nitrogen (BUN), creatinine, uric acid (UA), fasting plasma glucose (FPG), insulin, and glycated hemoglobin A_1c_ [HbA_1c_ (NGSP)] in a morning after 12-h fasting. All chemistries were measured at commercially available laboratories (SRL Inc. Laboratory, Fukuoka, Japan, and The Kyodo Igaku Laboratory, Fukuoka, Japan). SRL Inc. Laboratory measured serum Lp(a) by latex immunoassay (LIA) method (SEKISUI Medical, Tokyo, Japan) [15], apolipoproteins by turbidimetric immunoassay method (SEKISUI Medical, Tokyo, Japan), and directly measured RLP-C by an immuno-separation technique (using an immunoaffinity gel containing monoclonal antibodies to human apo B-100 and apo A-I) (MiNARis medical, Tokyo, Japan) [16]. Intra- and inter-assay coefficients of variation of RLP-C in the commercially available laboratory (SRL inc. Laboratory, Fukuoka, Japan) were 7.6% and 7.8%, respectively. Other chemicals were examined in The Kyodo Igaku Laboratory, Fukuoka, Japan.

Fasting blood samples were centrifuged within 1 h after the collection. Estimated glomerular filtration rate (eGFR) was calculated using the Modification of Diet in Renal Disease (MDRD) study equation modified with a Japanese coefficient [17]. Homeostasis model assessment of insulin resistance (HOMA-IR) was calculated from FPG and insulin levels [FPG (mg/dL) × insulin (μU/mL)/405] as a marker of IR [18]. Estimated RLP-C levels were defined as the following formula [total cholesterol – (LDL-C) − (HDL-C)]. The cut-off value of RLP-C was defined as greater than 7.5 mg/dL or not according to guidance of the SRL Inc. Laboratory [19].

Eating pattern was evaluated by a brief-type self-administered diet history questionnaire (BDHQ) [20]. The BDHQ is a four-page, fixed-portion questionnaire that asks about the consumption frequency of selected foods, but not about portion size, to estimate the dietary intake of fifty-eight food and beverage items during the preceding month. To facilitate reading and completion for the elderly, we used a large-print version, which increased the size to ten pages but that contained no other changes to structure or content. Details of the BDHQ’s structure, method of calculating dietary intake, and validity for food group and nutrient intakes among the adult population (31–76 years) have been described elsewhere [21, 22]. Briefly, the BDHQ consists of the following five sections: (i) intake frequency of food and non-alcoholic beverage items; (ii) daily intake of rice and miso soup; (iii) frequency of drinking and amount per drink for alcoholic beverages; (iv) usual cooking methods; and (v) general dietary behavior. Food and beverage items contained in the BDHQ were selected from foods commonly consumed in Japan, mainly from a food list used in the National Health and Nutrition Survey of Japan, while standard portion sizes were derived from several recipe books for Japanese dishes [22]. Information on dietary supplements was obtained only for total frequency of use, without specific names or types and quantity of the supplements. Estimates of the intake for fifty-eight food and beverage items were calculated using an ad hoc computer algorithm for the BDHQ [22].

### Statistical analysis

Because of skewed distributions, the natural logarithmic transformation was performed for RLP-C, Lp(a), insulin, HOMA-IR, and triglycerides. Mean values, upper and lower 95% confidence limits, were exponentiated and presented geometric mean ± standard deviation (SD), where the SD was approximated as the difference of the exponentiated confidence limits divided by 3.92, the number of SD in a 95% confidence interval for normally distributed data. Chi-square tests were used for evaluation of categorical parameters. Uni- and multiple linear regression analyses adjusted for age and sex were used. Using some significant factors from multivariate linear regression analysis adjusted for age and sex, we performed the multiple stepwise regression analysis to see the strength and independency for estimated RLP-C. ROC analysis was performed to evaluate diagnostic ability of estimated RLP-C and HOMA-IR on dichotomized RLP-C (≥ 7.5 mg/dL or < 7.5 mg/dL). The C-statistics was reported as a measure of diagnostic ability, and Youden’s index was used to identify cut-off point on the ROC curve, which was defined as the maximum of (sensitivity + specificity-1).

*P* values < 0.05 were considered statistically significant. All statistical analyses were performed using SPSS version 26.0 (IBM Inc., Chicago, IL, USA).

## Results

### Estimated and directly measured RLP-C and eating pattern

**Fig. 1:**
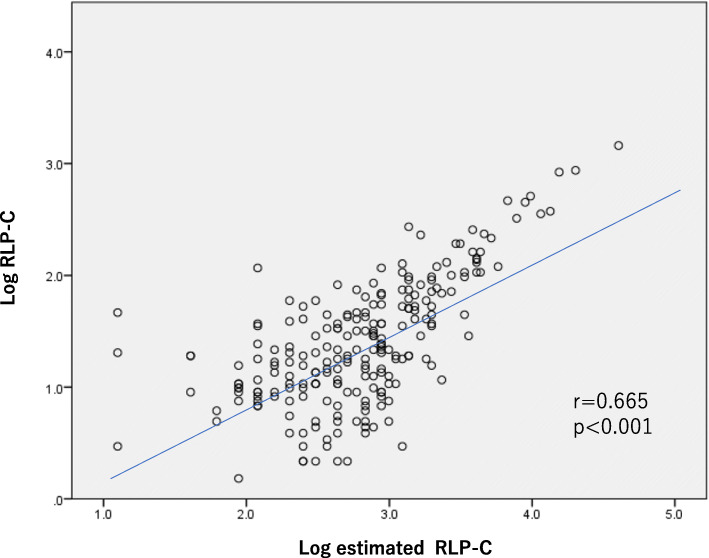
The correlation between estimated RLP-C and RLP-C

**Fig. 2:**
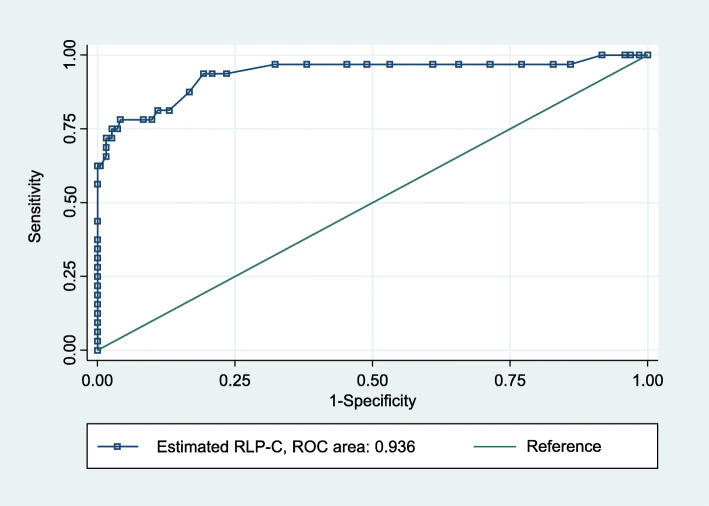
Optimal estimated RLP-C cut-off values for RLP-C in ROC analysis

Characteristics of 225 subjects are presented in Table 1. There was no significant gender difference between directly measured RLP-C and estimated RLP-C. Lp(a) was significantly higher (*p* < 0.05) in females than in males. The total daily energy intake of the study subjects in the present study was 1845 kcal, the daily calorie intake from carbohydrate, protein and fat were 238.4 g, 72 g, and 56.1 g, respectively. Figure 1 shows the significant correlation between RLP-C and estimated RLP, although these data did not show the same absolute value. Pearson correlation coefficient was very high (*r* = 0.665; *p* < 0.001). Figure 2 shows ROC curve for estimated RLP-C with C-statistics of 0.936. The calculated cut-off value was 22.0 mg/dL with estimated RLP-C.

**Table 1:** Characteristics of the subjects

	Male (*n* = 94)	Female (*n* = 131)	Total (*n* = 225)
Age (years)	**69.2** ± **7.2**	**68.9** ± **8.3**	**69.0** ± **7.9**
Body mass index (kg/m^2^)	**24.3** ± **3.2**	**23.9** ± **3.8**	**24.1** ± **3.5**
Waist circumference (cm)	**86.2** ± **8.9**	**84.2** ± **9.4**	**85.0** ± **9.3**
Systolic blood pressure (mmHg)	**139.1** ± **18.9**	**136.1** ± **19.0**	**137.4** ± **18.9**
Diastolic blood pressure (mmHg)	**78.2** ± 11.3*	**75.2** ± **9.7**	**76.5** ± **10.5**
Blood urea nitrogen (mg/dL)	**16.1** ± **4.2**	**16.2** ± **4.7**	**16.1** ± **4.5**
Creatinine (mg/dL)	**0.83** ± 0.16***	**0.68** ± **0.23**	**0.74** ± **0.21**
Estimated GFR (mL/min/1.73m^2^)	**73.6** ± **15.9**	**69.7** ± **17.1**	**71.3** ± **16.7**
Uric acid (mg/dL)	**5.9** ± 1.2***	**4.8** ± **1.0**	**5.2** ± **1.1**
Fasting plasma glucose (mg/dL)	**102.7** ± 15.6***	**92.3** ± **10.6**	**96.7** ± **13.9**
Insulin (μU/mL)	**4.7 (1.1–19.9)**	**4.4 (1.1–17.5)**	**4.5 (1.1–19.9)**
HOMA-IR^†^ (min-max)	**1.17 (0.27–5.94)**	**0.99 (0.24–4.74)**	**1.07 (0.24–5.94)**
Hemoglobin A_1c_ (NGSP%)	**5.63** ± **0.42**	**5.58** ± **0.37**	**5.60** ± **0.39**
Total cholesterol (mg/dL)	**187.1** ± **31.5**	**208.7** ± 35.4***	**199.6** ± **35.4**
LDL-C (mg/dL)	**105.5** ± **26.7**	**120.7** ± 30.7***	**114.3** ± **30.0**
HDL-C (mg/dL)	**62.1** ± 16.7**	**68.9** ± **16.4**	**66.0** ± **16.8**
Triglycerides (mg/dL) ^†^ (min-max)	**94.6 (32–338)**	**88.0 (28–306)**	**90.7 (28–338)**
RLP-C (mg/dL)^a^ (min-max)	**3.9 (1.2–18.9)**	**4.1 (1.4–23.6)**	**4.0 (1.2–23.6)**
Estimated RLP-C (mg/dL)^a^ (min-max)	**16.6 (3–74)**	**16.3 (3–100)**	**16.4 (3–100)**
Lipoprotein (a) (mg/dL)^a^ (min-max)	**6.2 (1–63)**	8.8 (1–107)*	**7.6 (1–107)**
Apolipoprotein A-I (mg/dL)	**157.0** ± **28.2**	**169.4** ± 29.5**	**164.2** ± **29.6**
Apolipoprotein B (mg/dL)	**86.3** ± **10.0**	**91.7** ± 20.0*	**89.4** ± **19.7**
Apolipoprotein C-III (mg/dL)	**9.57** ± **3.57**	**9.64** ± **2.69**	**9.61** ± **3.08**
Apolipoprotein E (mg/dL)	**4.15** ± **1.02**	**4.83** ± 1.18***	**4.55** ± **1.16**
Smoking (%, yes)	23.4 (*n*= 22)***	**0.8 (*n*= 1)**	**10.2 (*n*= 23)**
Alcohol (%, yes)	63.8 (*n*= 60)***	**9.2 (*n*= 12)**	**32.0 (*n*= 72)**
HT medication (%, yes)	71.2 (*n*= 67)*	**58.8 (*n*= 77)**	**64.0 (*n*= 144)**
DM medication (%, yes)	**14.9 (*n*= 14)**	**9.2 (*n*= 12)**	**11.6 (*n*= 26)**
DL medication (%, yes)	**46.8 (*n*= 44)**	59.5 (*n*= 78)*	**54.2 (*n*= 122)**
Energy (kcal/day)	**2078.3** ± **516.9**	**1678.7** ± **471.0**	**1845.6** ± **527.9**
Animal protein (g/day)	**44.2** ± **19.0**	**40.5** ± **21.2**	**42.1** ± **20.4**
Vegetable protein (g/day)	**32.0** ± **9.7**	**28.4** ± **8.4**	**29.9** ± **9.1**
Animal fat (g/day)	**28.1** ± **12.5**	**25.5** ± **12.4**	**26.6** ± **12.5**
Vegetable fat (g/day)	**30.2** ± **10.3**	**29.0** ± **11.7**	**29.5** ± **11.1**
Carbohydrate (g/day)	**264.8** ± **76.3**	**219.4** ± **59.3**	**238.4** ± **70.5**
Saturated fatty acid (g/day)	**15.4** ± **6.5**	**14.6** ± **6.5**	**14.9** ± **6.5**
Monounsaturated fatty acid (g/day)	**20.6** ± **7.2**	**19.4** ± **8.0**	**19.9** ± **7.6**
Polyunsaturated fatty acid (g/day)	**14.6** ± **4.4**	**13.4** ± **4.8**	**13.9** ± **4.7**

*SE* standard error, *HOMA*-*IR* homeostasis model assessment of insulin resistance, *LDL*-*C* low-density lipoprotein cholesterol, *HDL*-*C* high-density lipoprotein cholesterol, *RLP*-*C* remnant-like particle cholesterol, *HT* hypertension, *DM* diabetes, *DL* dyslipidemia

Data are means ± standard deviation or percentage, unless otherwise indicated

^a^Log-transformed values were used for the statistical calculation and reconverted to antilogarithm forms in the tables

*p < 0.05; **p < 0.01; ***p < 0.001

### Estimated RLP-C and nutrient intake

Table 2 shows the results of uni- and multivariate analyses adjusted for age and sex for correlates of estimated RLP-C. In multivariate analysis, insulin (*p* = 0.01), HOMA-IR (*p* = 0.01), total cholesterol (*p* = 0.007), HDL-C (*p* = 0.009; inversely), triglycerides (*p* < 0.001), RLP-C (*p* < 0.001), apo B (*p* < 0.001), apo C-III (*p* < 0.001), apo E (*p* < 0.001), vegetable fat (*p* < 0.001; inversely), saturated fatty acid (*p* = 0.009), monounsaturated fatty acid (*p* = 0.009; inversely), and polyunsaturated fatty acid (*p* = 0.018; inversely) were significantly associated with estimated RLP-C. Using the significant factors detected by multivariate analysis adjusted for age and sex in Table 2, we performed the multiple stepwise regression analysis. Eventually, RLP-C (*p* < 0.001) was the strongest correlate for estimated RLP-C and age was the second (*p* < 0.001). And, the significances of apolipoproteins C-III (*p* < 0.001) and E (*p* = 0.011), triglycerides (*p* = 0.007), and vegetable fat (*p* = 0.002; inversely) were still remained (Table 3).

**Table 2:** Association between estimated RLP-C and parameters

Parameters	Univariate			Multivariate (adjusted for age and sex)		
	β	SE	***p*** value	β	SE	***p*** value
Age	0.017	0.005	**< 0.001**			
Sex (Male = 0, Female = 1)	− 0.018	0.077	0.811			
Body mass index	0.018	0.011	0.088			
Waist circumference	0.006	0.004	0.123			
Systolic blood pressure	0.005	0.002	**0.014**	0.003	0.002	0.092
Diastolic blood pressure	0.004	0.004	0.248			
Blood urea nitrogen	0.009	0.008	0.314			
Creatinine	0.339	0.176	0.055			
Estimated GFR	− 0.003	0.002	0.257			
Uric acid	0.033	0.033	0.325			
Fasting plasma glucose	0.004	0.033	0.138			
Insulin^a^	0.152	0.059	**0.011**	0.149	0.058	**0.010**
HOMA-IR^a^	0.144	0.054	**0.008**	0.139	0.053	**0.010**
Hemoglobin A_1c_ (NGSP)	0.090	0.097	0.350			
Total cholesterol	0.002	0.001	**0.033**	0.003	0.001	**0.007**
LDL-C	− 0.002	0.001	0.117			
HDL-C	− 0.006	0.002	**0.008**	− 0.006	0.002	**0.009**
Triglycerides^a^	0.786	0.059	**< 0.001**	0.774	0.058	**< 0.001**
RLP-C^a^	0.676	0.051	**< 0.001**	0.664	0.050	**< 0.001**
Lipoprotein (a)^a^	− 0.082	0.034	**0.018**	− 0.063	0.035	0.070
Apolipoprotein A-I	0.001	0.001	0.489			
Apolipoprotein B	0.007	0.002	**< 0.001**	0.008	0.002	**< 0.001**
Apolipoprotein C-III	0.099	0.010	**< 0.001**	0.105	0.010	**< 0.001**
Apolipoprotein E	0.207	0.029	**< 0.001**	0.229	0.029	**< 0.001**
Smoking	− 0.166	0.124	0.183			
Alcohol	0.153	0.080	0.058			
HT medication	0.066	0.037	0.075			
DM medication	− 0.076	0.061	0.216			
DL medication	0.034	0.040	0.405			
Energy	− 0.001	0.001	0.017			
Animal protein	0.001	0.002	0.855			
Vegetable protein	− 0.007	0.004	0.105			
Animal fat	− 0.003	0.003	0.259			
Vegetable fat	− 0.012	0.003	**< 0.001**	− 0.012	0.003	**< 0.001**
Carbohydrate	0.001	0.001	0.314			
Saturated fatty acid	0.017	0.006	**0.004**	0.015	0.006	**0.009**
Monounsaturated fatty acid	− 0.017	0.005	**0.008**	− 0.013	0.005	**0.009**
Polyunsaturated fatty acid	− 0.017	0.008	**0.030**	− 0.019	0.008	**0.018**

*SE* standard error, *HOMA*-*IR* homeostasis model assessment of insulin resistance, *LDL*-*C* low-density lipoprotein cholesterol, *HDL*-*C* high-density lipoprotein cholesterol, *RLP*-*C* remnant-like particle cholesterol, *HT* hypertension, *DM* diabetes, *DL* dyslipidemia

^a^Log-transformed values were used for the statistical calculation and reconverted to antilogarithm forms in the tables

**Table 3:** Multiple stepwise linear regression analysis between estimated RLP-C

Parameters	β	S.E.	*p* value
RLP-C^a^	0.676	0.051	**< 0.001**
Age	0.014	0.004	**< 0.001**
Apolipoprotein C-III	0.052	0.011	**< 0.001**
Vegetable fat	− 0.007	0.002	**0.002**
Triglycerides^a^	0.314	0.115	**0.007**
Apoprotein E	0.089	0.027	**0.011**

*SE* standard error, *RLP*-*C* remnant-like particle cholesterol

^a^Log-transformed values were used for the calculation and reconverted to anti-logarithm forms

### Directly measured RLP-C and nutrient intake

Table 4 shows the results of uni- and multivariate analyses adjusted for age and sex for correlates of directly measured RLP-C. In multivariate analysis, BMI (*p* = 0.005), waist circumference (*p* = 0.013), systolic (*p* = 0.043) and diastolic (*p* = 0.016) BPs, uric acid (*p* = 0.007), insulin (*p* < 0.001), HOMA-IR (*p* < 0.001), total cholesterol (*p* < 0.001), LDL-C (*p* < 0.001), HDL-C (*p* < 0.001; inversely), triglycerides (*p* < 0.001), RLP-C (*p* < 0.001), Lp(a) (*p* = 0.023), apo B (*p* < 0.001), apo C-III (*p* < 0.001), apo E (*p* < 0.001), and vegetable fat (*p* = 0.032; inversely) were significantly associated with directly measured RLP-C. Using the significant factors detected by multivariate analysis adjusted for age and sex in Table 4, we performed the multiple stepwise regression analysis. Eventually, RLP-C (*p* < 0.001) was the strongest correlate for directly measured RLP-C and triglycerides was the second (*p* < 0.001). The significances of total cholesterol (*p* < 0.001), Lp(a) (*p* < 0.001; inversely), apolipoproteins B (*p* < 0.001) and E (*p* = 0.006), vegetable fat (*p* = 0.022; inversely), and HOMA-IR (*p* = 0.027; inversely) were still remained (Table 5).

**Table 4:** Association between directly measured RLP-C and parameters

Parameters	Univariate			Multivariate (adjusted for age and sex)		
	β	SE	***p***-value	β	SE	***p*** value
Age	0.005	0.005	0.263			
Sex (Male = 0, Female = 1)	0.060	0.075	0.423			
Body mass index	0.029	0.010	**0.006**	0.030	0.010	**0.005**
Waist circumference	0.010	0.004	**0.016**	0.010	0.004	**0.013**
Systolic blood pressure	0.004	0.002	**0.031**	0.004	0.002	**0.043**
Diastolic blood pressure	0.007	0.004	**0.041**	0.009	0.004	**0.016**
Blood urea nitrogen	− 0.011	0.008	0.185			
Creatinine	0.332	0.173	0.057			
Estimated GFR	− 0.005	0.002	**0.024**	− 0.005	0.002	0.061
Uric acid	0.071	0.032	**0.028**	0.093	0.034	**0.007**
Fasting plasma glucose	0.003	0.003	0.224			
Insulin^a^	0.277	0.056	< **0.001**	0.279	0.056	< **0.001**
HOMA-IR^a^	0.245	0.051	< **0.001**	0.252	0.052	< **0.001**
Hemoglobin A_1c_ (NGSP)	0.024	0.095	0.803			
Total cholesterol	0.006	0.001	< **0.001**	0.007	0.001	< **0.001**
LDL-C	0.006	0.001	< **0.001**	0.006	0.001	< **0.001**
HDL-C	− 0.008	0.002	< **0.001**	− 0.009	0.002	< **0.001**
Triglycerides^a^	1.027	0.037	< **0.001**	1.036	0.036	< **0.001**
RLP-C^a^	0.654	0.049	< **0.001**	0.676	0.050	< **0.001**
Lipoprotein (a)^a^	− 0.077	0.034	**0.023**	− 0.080	0.035	**0.023**
Apolipoprotein A-I	0.001	0.001	0.732			
Apolipoprotein B	0.016	0.002	< **0.001**	0.017	0.002	< **0.001**
Apolipoprotein C-III	0.107	0.010	< **0.001**	0.109	0.010	< **0.001**
Apolipoprotein E	0.213	0.029	< **0.001**	0.224	0.030	< **0.001**
Smoking	− 0.172	0.122	0.160			
Alcohol	0.080	0.079	0.314			
HT medication	0.008	0.036	0.824			
DM medication	− 0.015	0.061	0.803			
DL medication	0.012	0.040	0.767			
Energy	− 0.007	0.001	0.297			
Animal protein	− 0.001	0.002	0.763			
Vegetable protein	− 0.007	0.004	0.094			
Animal fat	− 0.001	0.003	0.744			
Vegetable fat	− 0.007	0.003	**0.028**	− 0.007	0.003	0.032
Carbohydrate	0.001	0.001	0.353			
Saturated fatty acid	− 0.007	0.005	0.156			
Monounsaturated fatty acid	− 0.007	0.005	0.156			
Polyunsaturated fatty acid	− 0.013	0.008	0.114			

*SE* standard error, *HOMA*-*IR* homeostasis model assessment of insulin resistance, *LDL*-*C* low-density lipoprotein cholesterol, *HDL*-*C* high-density lipoprotein cholesterol, *RLP*-*C* remnant-like particle cholesterol, *HT* hypertension, *DM* diabetes, *DL* dyslipidemia

^a^Log-transformed values were used for the statistical calculation and reconverted to antilogarithm forms in the tables

**Table 5:** Multiple stepwise linear regression analysis between directly measured RLP-C and parameters

Parameters	β	S.E.	*p* value
Estimated RLP-C^†^	0.562	0.048	< **0.001**
Triglycerides^†^	0.934	0.010	< **0.001**
Total cholesterol	0.014	0.001	< **0.001**
Lipoprotein (a)^a^	− 0.040	0.011	< **0.001**
HDL-C	− 0.013	0.002	< **0.001**
Apoprotein B	0.013	0.002	< **0.001**
Apoprotein E	0.032	0.011	**0.006**
Vegetable fat	− 0.002	0.001	**0.022**
HOMA-IR^a^	− 0.039	0.017	**0.027**

*SE* standard error, *HOMA*-*IR* homeostasis model assessment of insulin resistance, *HDL*-*C* high-density lipoprotein cholesterol, *RLP*-*C* remnant-like particle cholesterol

*R*^2^ = 0.923

^a^Log-transformed values were used for the calculation and reconverted to anti-logarithm forms

## Discussion

In the present study, we demonstrated for the first time that estimated RLP-C levels using conventional lipid profiles may substitute for directly measured RLP-C and that these levels were independently and inversely associated with vegetable fat intake in the community-dwelling Japanese population. Estimated RLP-C was much easier to measure at lower cost than directly measured RLP-C.

Apparently in Fig. 1, the correlation between RLP-C and estimated RLP was so strong. Pearson correlation coefficient was very high (*r* = 0.665). To the best of our knowledge, there have been no reports dealing with examinations using estimated RLP-C. In the present study, the mean levels of RLP-C in males and females were 3.9 mg/dL and 4.1 mg/dL, respectively. These values were similar to those reported in Japanese control subjects [19], but much lower than those 8.0 mg/dL for males 6.8–7.2 mg/dL for females of participants reported in the Framingham Heart Study [2]. Although it was reported from Japan [3] that the risk of cardiovascular events increased in the high RLP-C group (> 4.7–5.1 mg/dL), we cannot comment on this issue because our study was not prospective one. When we classified RLP-C cut-off values into 2 groups (< 7.5 mg/dL vs. ≥ 7.5 mg/dL), the calculated value of estimated RLP-C by ROC curve was 22.0 mg/dL with the C-statistics of 0.936 (Fig. 2). The value may be useful for checking dangerous levels for estimated RLP-C without directly measured RLP-C.

Faridi et al. [23] suggested that estimate of RLP-C using basic lipid parameters correlated weakly with remnants measured by ultracentrifugation; however, their formula of estimated RLP-C was relatively complicated rather than ours.

Several investigations regarding to the relationship between RLP-C and apolipoproteins or nutrient intake were found [4, 5, 7–9]. As well as directly measured RLP-C, estimated RLP-C levels were significantly and positively associated with apolipoproteins. As a result of stepwise regression analysis (Table 3), apolipoproteins C-III and E were independent correlates for estimated RLP-C.

Our data also indicated that estimated RLP-C levels were significantly and independently associated with log-transformed triglycerides level (Table 3). As for RLP-C, Abbasi et al. [24] reported that RLP-C and triglycerides concentrations were elevated in nondiabetic, insulin-resistant, female volunteers. Although there were a small number of diabetic subjects (*n* = 26) in our population, RLP-C and triglycerides concentrations were also elevated in nondiabetic, insulin-resistant subjects. Although insulin or HOMA-IR failed to indicate the significance in the multiple stepwise regression analysis, insulin resistance or metabolic syndrome was potentially associated with RLP-C. Our previous study [25] suggested that the RLP-C level in the metabolic syndrome was very high at > 7.9 mg/dL, compared with the average Japanese value of 3.3 mg/dL.

The dietary data that were used for validation were compared with the results of the Japanese National Nutrition Survey in 2019 [12]. The results of the National Nutrition Survey in 2019 are shown in parentheses. The total daily energy intake of the study subjects in the present study was 1846 kcal (vs. 1849 kcal); the percentages of the daily calorie intake from carbohydrate, protein, and fat were 52% (vs. 56%), 16% (vs. 15%), and 27% (vs. 28%), respectively. Thus, the eating pattern in the subjects of the present study was similar to that reported in the results of the National Nutrition Survey. It is interesting to note that monounsaturated fatty acid and polyunsaturated fatty acid including vegetable fat were inversely associated with estimated RLP-C (Table 2). Although vegetable fat intake was the strongest associator in these nutrients, these inversely associations between RLP-C and the nutrients were supported by some investigators [26–28]. In Japan, most people eat soy protein as vegetable fat, and Japanese investigators demonstrated that soy protein isolate intake reduced remnant lipoproteins [10, 11]. Thus, it can be the first demonstration for the inverse significant association between RLP levels and vegetable fat intake in a general Japanese population.

## Limitations

There are several limitations in our study. First, the study design was cross-sectional. Thus, nothing conclusive can be stated with regard to the associations of estimated RLP-C and lipids or nutritional intake. We are planning future prospective studies to investigate the role of these markers in subjects with atherosclerosis. Second, the results from only a single blood test were used to evaluate the associations of estimated RLP-C and the lipids. The third limitation of the study is the relatively small sample size. While using multiple linear regression, there are around 40 independent variables in the model, which means the samples size should be at least 200–400. This study included a total of 225 individuals, which might bring the estimation bias. The fourth limitation is that estimated RLP-C is not accurate compared to the directly measured RLP-C, in which the simple formula is recently used to measure RLP-C cutoff values using total cholesterol, LDL-C, and HDL-C [6]. However, more importantly in the clinical settings, it should be clarified in the future studies that both of estimated and directly measured RLP-C are therapeutic targets, or that either of them will be enough to reduce cardiovascular events. The fifth limitation is that because Uku town, an isolated island and a fishing community, is located in southwestern Japan, the study subjects may not represent a general population.

## Conclusions

In conclusion, we demonstrated that the estimated RLP-C levels using conventional lipid profiles may substitute for directly measured RLP-C and both of them were independently and inversely associated with vegetable fat intake in the community-dwelling Japanese population.

## Abbreviations

RLP-C: Remnant-like particle cholesterol
LDL-c: Low-density lipoprotein cholesterol
HDL-c: High-density lipoprotein cholesterol
ROC: Receiver operating characteristic
BMI: Body mass index
BP: Blood pressure
Lp(a): Lipoprotein(a)
UA: Uric acid
FPG: Fasting plasma glucose
HbA_1c_: Glycated hemoglobin A_1c_
eGFR: Estimated glomerular filtration rate
HOMA-IR: Homeostasis model assessment of insulin resistance
BDHQ: Brief-type self-administered diet history questionnaire
SD: Standard deviation
